# NRF-2/HO-1 Pathway-Mediated SHOX2 Activation Is a Key Switch for Heart Rate Acceleration by Yixin-Fumai Granules

**DOI:** 10.1155/2022/8488269

**Published:** 2022-09-26

**Authors:** Heng Zhang, Chen Chen, Yue Liu, Lu Ren, Jing Qi, Yang Yang, Wei Chen, Yingjia Yao, Xintong Cai, Zhuang Liu, Miao Hao, Lingkang Li, Zisu Deng, Mingyu Sun, Yongping Lu, Keyan Chen, Ping Hou

**Affiliations:** ^1^Liaoning University of Traditional Chinese Medicine, Shenyang 110000, China; ^2^Department of Cardiology, Affiliated Hospital of Liaoning University of Traditional Chinese Medicine, Shenyang 110000, China; ^3^Department of Cardiology, The Second Affiliated Hospital of Shenyang Medical University, Shenyang 110000, China; ^4^Northeastern University, Shenyang 110000, China; ^5^Department of NHC Key Laboratory of Reproductive Health and Medical Genetics, Liaoning Research Institute of Family Planning (The Affiliated Reproductive Hospital of China Medical University), Shenyang 110000, China; ^6^Department of Laboratory Animal Science, China Medical University, Shenyang 110000, China

## Abstract

Population aging has led to increased sick sinus syndrome (SSS) incidence; however, no effective and safe medical therapy has been reported thus far. Yixin-Fumai granules (YXFMs), a Chinese medicine granule designed for bradyarrhythmia treatment, can effectively increase SSS patients' heart rate. Senescence-induced sinoatrial node (SAN) degeneration is an important part of SSS pathogenesis, and older people often show high levels of oxidative stress; reactive oxygen species (ROS) accumulation in the SAN causes abnormal SAN pacing or conduction functions. The current study observed the protective effects of YXFMs on senescent SAN and explored the relationship between the NRF-2/HO-1 pathway, SHOX2, and T-type calcium channels. We selected naturally senescent C57BL/6 mice with bradycardia to simulate SSS; electrocardiography, Masson's trichrome staining, and DHE staining were used to assess SAN function and tissue damage. Immunofluorescence staining and Western blotting were used to assay related proteins. *In vitro*, we treated human-induced pluripotent stem cell-derived atrial myocytes (hiPSC-AMs) and mouse atrial myocyte-derived cell line HL-1 with D-galactose to simulate senescent SAN-pacemaker cells. CardioExcyte96 was used to evaluate the pulsatile function of the hiPSC-AMs, and the mechanism was verified by DCFH-DA, immunofluorescence staining, RT-qPCR, and Western blotting. The results demonstrated that YXFMs effectively inhibited senescence-induced SAN hypofunction, and this effect possibly originated from scavenging of ROS and promotion of NRF-2, SHOX2, and T-type calcium channel expression. *In vitro* experiment results indicated that ML385, si-*SHOX2*, LDN193189, and Mibefradil reversed YXFMs' effects. Moreover, we, for the first time, found that ROS accumulation may hinder SHOX2 expression; YXFMs can activate SHOX2 through the NRF-2/HO-1 pathway-mediated ROS scavenging and then regulate CACNA1G through the SHOX2/BMP4/GATA4/NKX2-5 axis, improve T-type calcium channel function, and ameliorate the SAN dysfunction. Finally, through network pharmacology and molecular docking, we screened for the most stable YXFMs compound that docks to NRF-2, laying the foundation for future studies.

## 1. Introduction

Sick sinus syndrome (SSS) is a disease primarily characterized by dysfunctional pace function and sinoatrial node (SAN) conducting issues. It is often a cause of pathology-based shifts within SAN along with SAN-encompassing tissue and is commonly noted among elderly people. SSS frequently shows as sinus bradycardia, atrioventricular block, sinus arrest, or other arrhythmias; it is also one of the most frequent causes of cardiogenic syncope and unexpected death [1]. Artificial pacemaker implantation can be the most effective treatment for SSS; however, the procedure is cost intensive with a high probability of postoperative atrial fibrillation. Moreover, conservative SSS treatment, mostly focused on the administration of atropine, isoprenaline, and other similar drugs, has serious side effects that can result in life-threatening malignant arrhythmias [2–4]. Scientifically validated traditional Chinese medicine modalities applicable to SSS treatment are needed urgently.

SAN degeneration changes are an important indicator of SSS pathogenesis [5]. Phase-4 automatic depolarization by pacemaker (P) cells within SAN is the key to maintaining the SAN autonomous rhythm. The cation influx of phase-4 automated P cell depolarization can be mainly mediated by hyperpolarization-activated cyclic nucleotide-gated cation channel 4 (HCN4) and also by T-type calcium channels. T-type calcium channels, widely distributed on P cells and potential pacing cells in the SAN, are mainly low-voltage-activated calcium channels, with rapid activation-deactivation ability. The activation threshold potential of these channels in P cells is close to the maximum repolarization potential (approximately −70 mV). T-type calcium channels participate within the developmental process for cardiac conduction systems. Moreover, *I*_Ca−T_ is the current generated by T-type calcium channel activation, which assists in the maintenance of the normal autonomous rhythm in the SAN. In cardiac conduction systems, P cells have stronger *I*_Ca−T_ current than nonpacing cells and blocking the *I*_Ca−T_ current leads to decreases in the heart rate [6–9]. Voltage-dependent T-type calcium channel subunit alpha-1G (*Cacna1g*) is a key gene encoding T-type calcium channels, and its expression in SAN P cells is 30 times higher than that in atrial working myocytes [10]. The disruption of *Cacna1g* causes *I*_Ca−T_ to disappear, leading to bradycardia and atrioventricular block and thus extending SAN recovery time [11, 12].

Short stature homeobox 2 (SHOX2), a transcription regulator highly expressed in the SAN, inhibits NK2 homeobox 5 (NKX2-5) expression by regulating the downstream bone morphogenetic protein 4 (BMP4) and GATA binding protein 4 (GATA4) and plays a key role in SAN development and differentiation [13–15]. In a study, *Shox2*-knockout mice died during pregnancy due to cardiac conduction system defects, including SAN and valve sinus hypoplasia accompanied by the loss of T-box transcription factor 3 and HCN4 expression and ectopic natriuretic peptide A, connexin 40, and NKX2-5 expression in the SAN; this suggested that in *Shox2*-knockout mice, SAN cells cannot differentiate into P cells but into working cardiomyocytes [15–17]. The regulatory effect of *Shox2* on downstream *Bmp4*, *Gata4*, and *Nkx2-5* expression aids in preserving *Hcn4* and *Cacna1g* expression, which is of great significance for the maintenance of SAN function and the inhibition of the transformation of P cells into working cardiomyocytes [18–21].

The SSS incidence is related to age: the older the population, the higher the risk, and the normal SAN function gradually declines with age [22, 23]. As an inevitable life process, senescence is regulated by various complicated mechanisms, in which oxidative stress is not only a condition but also a result of senescence; according to the oxidative stress hypothesis, senescence results from the decline in the body's antioxidant capacity, which leads to age-dependent impairment caused by reactive oxygen species (ROS) accumulation [24, 25]. The antioxidant system has multiple components, of which the nuclear factor-like 2/heme oxygenase 1 (NRF-2/HO-1) pathway is a key signaling pathway for oxidative stress alleviation. Under physiological conditions, NRF-2 is present in the cytoplasm in an inert state, bound to its inhibitory protein Kelch-like ECH-associated protein 1 (KEAP-1), through KEAP-1/cullin-3/ring-box-dependent ubiquitination. Under oxidative stress, NRF-2 becomes activated and dissociates from Keap-1 to enter the nucleus, combine with antioxidant responsive elements (AREs), and initiate HO-1 transcription. HO-1 is a crucial antioxidant enzyme involved in multiple life processes, and the NRF-2/HO-1 pathway is one of the most important parts of the endogenous antioxidant system in the body [26–28].

Yixin-Fumai granules (YXFMs) are marketed traditional Chinese medicine compound granules, developed for bradyarrhythmia treatment (national medicine permission number: Z21021261); they can improve the heart rate with a considerable therapeutic effect [29, 30]. Its main components are ginseng, ophiopogon, schisandra, astragalus, salvia, and *Ligusticum wallichii*; both of them have strong antioxidant effects. In a previous study, we found that YXFMs can inhibit ROS accumulation in the SAN of SSS mice by activating the NRF-2/HO-1 pathway and then regulate histone deacetylase 4 to improve HCN4 expression and ameliorate SAN function [31]. In the current study, we for the first time explored the relationship between the senescence-induced accumulation of ROS, the expression of SHOX2 and T-type calcium channels in the SAN, and the effects of the NRF-2/HO-1 pathway. Here, naturally senescent C57BL/6 mice (age > 18 months) with a considerably reduced heart rate (>20%) were used to simulated senescence-induced SSS and human-induced pluripotent stem cell-derived atrial myocytes (hiPSC-AMs) as well as mouse atrial myocyte-derived cell line HL-1 were used for electrophysiological experiments and mechanism verification. Finally, the most stable compound in YXFMs docking with NRF-2 was screened through network pharmacology and molecular docking.

## 2. Methods and Materials

### 2.1. Animals

In total, 12 senescent C57BL/6 mice (age > 18 months; both genders) with a signifying declination heart rate (>20%) were used to simulated senescence-induced SSS. These mice were randomly but equally divided between an SSS group and a YXFM group (*n* = 6 each). Moreover, 6 younger adult mice (age = 3 months; both genders) with a normal heart rate were included in the control group. Each YXFM mouse was administered YXFMs at 1 g/kg/day via gavage for 30 days, whereas each mouse in those other groups was administered equal amounts of pure water via gavage for 30 days (the dose of YXFMs was based on a previous study [31]). Following this timeframe, resting heart rates and R-R intervals of all mice were recorded through electrocardiography. Thereafter, the mice were euthanized using 150 mg/kg intraperitoneal (i.p.) sodium pentobarbital and their heart tissues were collected. This *in vivo* investigation was examined and approved by the Animal Care and Use Committee of China Medical University.

YXFMs (national medicine permission number: Z21021261) and mice (production license: SCXK (Liao) 2018-0001) used in this study were acquired through the Tasly Pharmaceutical Group (China) and Changsheng Biotechnology (China), respectively.

### 2.2. Masson's Trichrome Staining

The collected heart tissue was embedded in paraffin; the blocks were vertically sliced at the anatomical position of the SAN to obtain 4 *μ*m thick serial sections. After deparaffinizing and washing them, we stained nuclei by using Weigert's iron hematoxylin for 10 minutes. After another wash, the sections were stained using Masson's composite staining solution for 10 minutes. Next, the sections were soaked in 2% glacial acetic acid and then in 1% dodeca molybdophosphoric acid solution for 5 minutes for differentiation. Next, we stain the sections with aniline blue for 5 minutes and then soaked them in 0.2% glacial acetic acid without washing. Finally, sections were dehydrated in 95% ethanol, followed by absolute ethanol. Xylene and a neutral glue were applied for clearing and sealing, respectively.

Masson's composite staining solution and Weigert's iron hematoxylin were acquired through Solarbio Biotechnology (China).

### 2.3. Cell Culture

hiPSC-AMs and HL-1 cells were applied to simulate P cells [32, 33], used for the electrophysiological assay and mechanism research of YXFMs, respectively. Furthermore, the hiPSC-AMs were cultured in the cardiomyocyte culture medium, whereas the HL-1 cells remained grown within Dulbecco's modified Eagle's medium (DMEM)/F12 supplemented with 10% fetal bovine serum (FBS) and 1% penicillin/streptomycin. All cell-based cultures in this study were preserved at 37°C in a 5% CO_2_ atmosphere. Next, to induce senescence, these cells were treated of D-galactose (10 g/L) for 48 hours. This was followed by treatment with YXFMs (5 g/L), ML385 (5 *μ*M), LDN193189 (0.5 *μ*M), mibefradil (0.5 *μ*M), or SB4 (0.1 *μ*M) for 24 hours (the dose of YXFMs was based on the previous study, calculated according to the CCK-8 assay [31]).

We obtained the culture media for the hiPSC-AMs and cardiomyocytes from Help Stem Cell Innovations (China); the analysis certificate can be found in the supplementary files (available here), the HL-1 cells from Procell Life Science (China), the DMEM/F12 medium and penicillin/streptomycin solution from HyClone (USA), FBS from Sijiqing Biotechnology (China), and finally, D-galactose, ML385, SB4, LDN193189, and Mibefradil from Topscience (China).

### 2.4. siRNA Transfection and Reverse Transcription Quantitative Polymerase Chain Reaction (RT-qPCR)

To prepare an siRNA–lipid complex for transfection, siRNAs were incubated with Lipofectamine RNAiMAX transfection reagent diluted with Opti-MEM medium (final siRNA concentration = 20 nM) at ambient temperature for 5 min for 24-hour transfection. For RT-qPCR, total RNA was isolated using the TRIzol reagent and reverse transcribed to cDNA by using BeyoRT M-MuLV Reverse-Transcriptase (42°C/60 minutes; 70°C/5 minutes). Resulting cDNA was detected by using a fluorescent RT-qPCR kit, with the following thermocycling conditions: 94°C for 5 min, followed by 40 cycles of 94°C for 10 s, 60°C for 20 s, and 72°C for 30 s. Relative gene expression data were inspected through 2^−ΔΔ*Ct*^ methodology.

We procured Lipofectamine RNAiMAX transfection reagent, Opti-MEM medium, and TRIzol reagent from Invitrogen (USA), BeyoRT M-MuLV Reverse Transcriptase from Beyotime Biotechnology (China), and the fluorescent qPCR kit from Solarbio Biotechnology (China). Moreover, the siRNAs (Table 1) were synthesized in Ribo Biotechnology (China), whereas the PCR primers (Table 2) were synthesized in Wanlei Biotechnology (China).

### 2.5. ROS Assay

For *in vivo* experiments, ROS investigation was conducted through using dihydroethidium (DHE), procured through Beyotime Biotechnology (China). After quickly frozen, the SAN anatomical position of collected heart tissue was found for serial slice (vertical, 4 *μ*m) to prepare frozen sections, followed by the administration of DHE and incubation at 37°C/1 hour. Following a phosphate-buffered saline (PBS) wash step, sections were examined under a fluorescence microscope.

Regarding *in vitro* experiments, ROS investigation had been conducted through using 2,7-dichlorodihydrofluorescein diacetate (DCFH-DA), procured through Beyotime Biotechnology (China). Cultured HL-1 cells were seeded onto a cell culture dish for laser confocal microscopy, followed by the administration of DCFH-DA and incubation at 37°C/1 hour. Following a PBS wash step, cells were examined under a confocal laser microscope and their fluorescence intensity was calculated.

### 2.6. Electrophysiological Assay

Frozen hiPSC-AMs were thawed using the cardiomyocyte thawing solution, after which were seeded onto a CardioExcyte 96 Sensor Plate coated with the cardiomyocyte plating solution at a density of 5 × 10^4^ cells/well. Next, the culture was maintained in the cardiomyocyte culture medium for 12 days. After grouping the cells and administering the various treatments, the CardioExcyte 96 system was used to assay the beat rate and field potential duration of the hiPSC-AMs.

This CardioExcyte 96 Sensor Plate was obtained from Nanion Technologies (Germany), and the cardiomyocyte thawing solution and cardiomyocyte plating solution were obtained from Help Stem Cell Innovation (China).

### 2.7. Immunofluorescence Staining

The process of immunofluorescence staining was employed as a way to assay NRF-2, visinin-like 1 (VSNL1), and CACNA1G expression in the SAN of mice in addition to NRF-2, SHOX2, and CACNA1G expression in HL-1 cells. For *in vivo* experiments, the collected heart tissue was embedded in paraffin and the anatomical position of the SAN was found for serial slice (vertical, 4 *μ*m). The paraffin sections were then dewaxed/dried and used in the antigen-retrieving process. Following that step, each of the sections was 1% bovine serum albumin (BSA) blockaded (60 minutes), the primary antibodies against VSNL1 (1 : 1000 dilution) and CACNA1G (1 : 200 dilution) or NRF-2 (1 : 200 dilution) were added, and then, the sections were placed in a humid box and incubated overnight (4°C). Following a wash, those AlexaFluor488-conjugated AffiniPure goat anti-chicken IgY (1 : 500 dilution) and CoraLite594- or CoraLite488-conjugated AffiniPure goat anti-rabbit IgG (1 : 500 dilution) were added, followed by incubation (37°C/1 hour). Following a separate wash, Antifade mounting medium with DAPI was added and the sections were placed for observation through a fluorescence microscope or laser scanning confocal microscope.

Regarding *in vitro* experiments, HL-1 cells were seeded onto a cell culture dish for laser confocal microscopy. Following culturing/treating, cells were fixed using 4% paraformaldehyde for 20 min and permeabilized with Triton X-100 for 20 min for the NRF-2 and SHOX2 assay. In contrast, no permeabilization was applied for the CACNA1G assay. Post-PBS wash, 1% BSA was added for blocking (60 min), followed by the addition of primary antibodies against NRF-2 (1 : 200 dilution), SHOX2 (1 : 200 dilution), or CACNA1G (1 : 200 dilution). Thereafter, dishes were inserted within a humid box and were then kept incubated overnight (4°C). Following a wash, CoraLite594- or CoraLite488-conjugated AffiniPure goat anti-rabbit IgG (1 : 500 dilution) was added, followed with an incubating step (37°C/60 minutes). Following a further wash, antifade mounting medium with DAPI was added and the cells were studied under a laser confocal microscope.

We purchased VSNL1 polyclonal antibody and AlexaFluor488-conjugated AffiniPure goat anti-chicken IgY from Abcam (USA); SHOX2 Polyclonal Antibody from Bioss (China); NRF-2 polyclonal antibody, CACNA1G polyclonal antibody, and CoraLite488- and CoraLite594-conjugated AffiniPure Goat anti-rabbit IgG from Proteintech (China); and 4% paraformaldehyde, Triton X-100, and antifade mounting medium with DAPI from Beyotime Biotechnology (China).

### 2.8. Western Blotting

Western blotting was used to assay the expression of SHOX2 in the SAN of mice and that of SHOX2-related proteins (SHOX2, BMP4, GATA4, and NKX2-5) in HL-1 cells. After extraction and quantifying, protein contents collected from mice and HL-1 cells were segregated by sodium dodecyl sulfate polyacrylamide gel electrophoresis (SDS-PAGE). Afterwards, the proteomic content was transported onto polyvinylidene difluoride (PVDF) membranes within Tris-glycine transfer buffer. Then, the membrane was blocked using 5% skimmed milk in TBST at ambient temperature (60 minutes). Following single TBST wash, membranes were placed into incubation with primary antibodies (1 : 500 dilution) against SHOX2, BMP4, GATA4, NKX2-5, and *β*-tubulin at 4°C overnight. Then, following another TBST-wash, membranes continued incubation with HRP-conjugated AffiniPure goat anti-rabbit IgG (1 : 8000 dilution) at ambient temperature (60 minutes), re-TBST-washed, and observed using BeyoECL Star Chemiluminescence kit.

We procured BMP4, GATA4, *β*-tubulin polyclonal antibodies, and HRP-conjugated AffiniPure goat anti-rabbit IgG through Wanlei Biotechnology (China); SHOX2 and NKX2-5 polyclonal antibodies from Proteintech (China); and SDS-PAGE precast gel, electrophoresis buffer, transfer buffer, protein marker, and BeyoECL Star Chemiluminescence Kit from Beyotime Biotechnology (China).

### 2.9. Network Pharmacology and Molecular Docking

Compounds and potential targets of ginseng, ophiopogon, schisandra, astragalus, salvia, and *L. wallichii* were obtained from the BATMAN-TCM database (http://bionet.ncpsb.org.cn/batman-tcm/). Next, by using the UniProt database (https://www.uniprot.org/), we matched proteins to their correct target genes. Furthermore, Cytoscape 3.7.2 was also made use of to build a YXFMs–herb–compound–target network, and then, NRF-2 was selected to build an herb–compound–NRF-2 network. Next, the cytoHubba plug in was used to calculate the degree value of the compounds interacting with NRF-2 and three compounds with the highest degree value were selected for molecular docking. The PubChem (https://pubchem.ncbi.nlm.nih.gov/), along with the software program Chem 3D, was used to obtain the structural formula of selected compounds and create the relevant 3D structure. Then, the NRF-2 domain was transferred from PDB (http://www.rcsb.org/). PyMOL was next employed as a way to remove water and phosphate in the proteins, and AutoDockTools 1.5.6 was then used to find the active pocket. Finally, we ran the Vina script to be able to assess and calculate the molecular binding energy and also to present the molecular docking results. Simultaneously, we ran Discovery Studio 2019 to find docking sites and calculate LibDockScore for flexible docking as well as PyMOL to display the molecular docking conformation; here, if binding energy of the ligand < 0, the receptors bind spontaneously, and if Vina binding energy ≤ −5.0 kcal · mol^−1^ and LibDockScore > 100, they form a stable docking. The molecular docking results of ligand–receptor complexes are displayed in 3D and 2D to evaluate the reliability of bioinformatics analysis and prediction.

### 2.10. Statistical Analysis

Each of the assays was replicated thrice for accuracy. Datasets were assessed through SPSS (version 26.0), reflecting means ± standard deviation. Statistical contrasts were then executed through one-way ANOVA. *P* < 0.05 was deemed to confer statistically significant results.

## 3. Results

### 3.1. YXFMs Enhanced SAN Roles within SSS Mice

**Figure 1:**
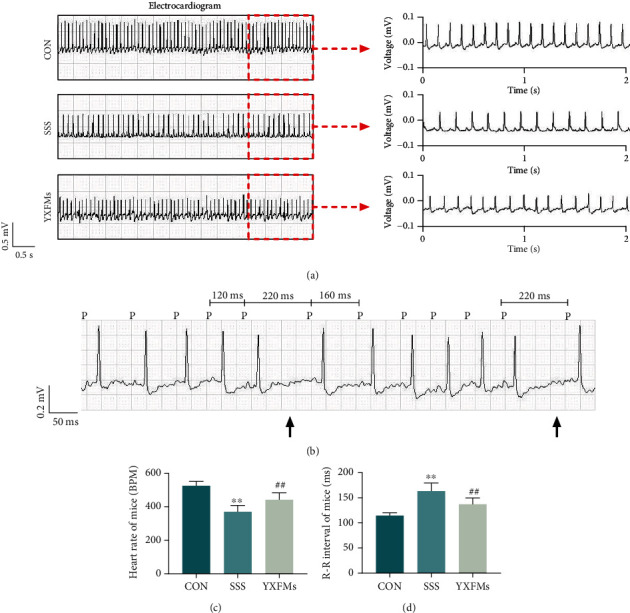
YXFMs enhanced SAN roles within SSS mice. (a) Electrocardiograms. (b) Wenckebach's sinoatrial block in SSS mice. (c, d) Comparative analyses for the resting heart rate and R-R intervals. Black arrows represent the dropped P-QRS-T complex. ^∗∗^*P* < 0.01 in comparison to the CON group, ^##^*P* < 0.01 in comparison to the SSS group. CON: control group; SSS: sick sinus syndrome group; YXFMs: Yixin-Fumai granule-administered group.

The resting heart rate and R-R intervals of mice in each group obtained via electrocardiography were employed for assessing SAN function. Results proved that the resting heart rate within the SSS group was substantially reduced in comparison to that within the control group and that R-R intervals were significantly prolonged (Figures 1(a), 1(c), and 1(d); ^∗∗^*P* < 0.01), and some mice shows Wenckebach's sinoatrial block phenomenon (Figure 1(b)): the P-P interval shortened gradually, the P-QRS-T complex dropped after the fourth P-P interval, the fifth P-P interval is 2 times shorter than the fourth P-P interval, and the sixth P-P interval was lengthier than the fourth P-P interval. These results suggested that senescence leads to SAN dysfunction. Contrasted with the SSS group, the resting heart rate/R-R intervals within YXFMs group improved significantly (Figures 1(a), 1(c), and 1(d); ^##^*P* < 0.01), and the sinoatrial block phenomenon disappeared as well. Such dataset outcomes suggested that YXFMs can notably enhance SAN activities within SSS mice.

### 3.2. YXFMs Alleviate SAN Fibrosis and ROS Accumulation in SSS Mice

**Figure 2:**
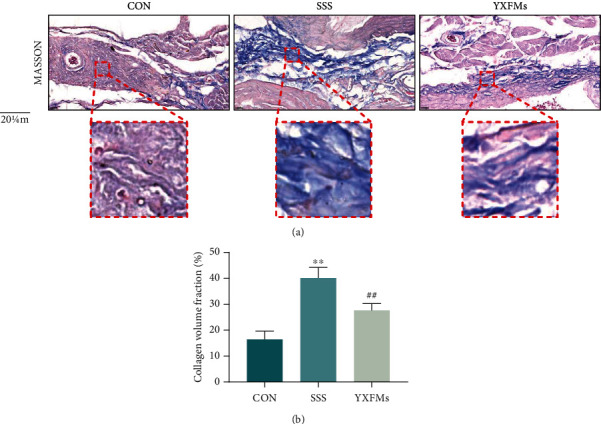
YXFMs alleviate SAN fibrosis within SSS mice. (a) Masson's trichrome staining (scale = 20 *μ*m). (b) Collagen volume fraction analysis. ^∗∗^*P* < 0.01 in comparison to the CON group, ^##^*P* < 0.01 in comparison to the SSS group. CON: control group; SSS: sick sinus syndrome group; YXFMs: Yixin-Fumai granule-administered group.

**Figure 3:**
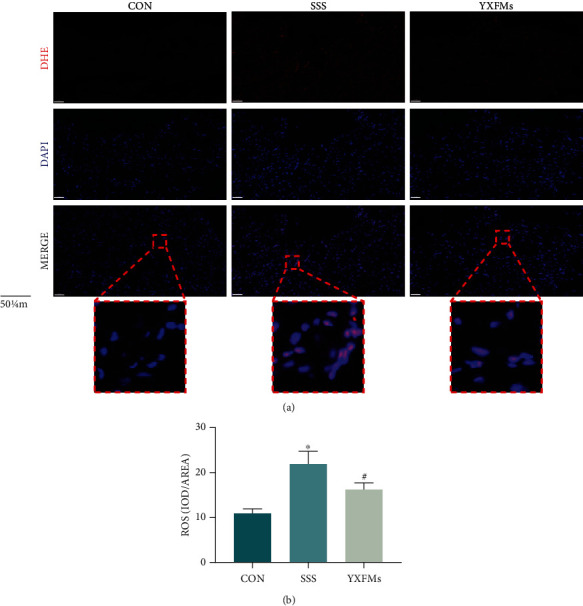
YXFMs alleviate SAN ROS accumulation within SSS mice. (a) DHE staining for ROS assay (scale = 50 *μ*m). (b) Comparative analyses for DHE staining. ^∗^*P* < 0.05 in comparison to the CON group, ^#^*P* < 0.05 in comparison to the SSS group. CON: control group; SSS: sick sinus syndrome group; YXFMs: Yixin-Fumai granule-administered group.

SAN fibrosis and ROS accumulation are the important characteristics of SAN degeneration. Masson's trichrome staining and collagen volume fraction (CVF) were used to analyze SAN fibrosis in the SSS mice; moreover, DHE staining was used to evaluate the ROS content in mouse SAN. The Masson's trichrome staining results demonstrated that compared with the CON group, more blue collagen fibers infiltrated into the SAN of the SSS group; however, after YXFMs treatment, the blue collagen fibers in the SAN of the YXFMs group were significantly fewer than those in the SAN of the SSS group. The CVF analysis results showed that the degree of SAN fibrosis in the SSS group was higher than that in the CON group (Figures 2(a) and 2(b); ^∗∗^*P* < 0.01), whereas the degree of SAN fibrosis in the YXFM group was significantly improved compared with that in the SSS group (Figures 2(a) and 2(b); ^##^*P* < 0.01). These results suggested that YXFMs can effectively alleviate senescence-induced SAN fibrosis. In addition, the DHE staining results showed that compared with the CON group, the red fluorescence intensity of the SAN in the SSS group mice increased significantly (Figures 3(a) and 3(b); ^∗^*P* < 0.05), demonstrating ROS accumulation in the SAN. However, after treatment with YXFMs, the red fluorescence in the SAN of the YXFM group was significantly lower than that in the SAN of the SSS group (Figures 3(a) and 3(b); ^#^*P* < 0.05), all suggesting that YXFMs can effectively alleviate senescence-induced SAN ROS accumulation.

### 3.3. YXFMs Alleviate NRF-2 Expression in SSS Mice

**Figure 4:**
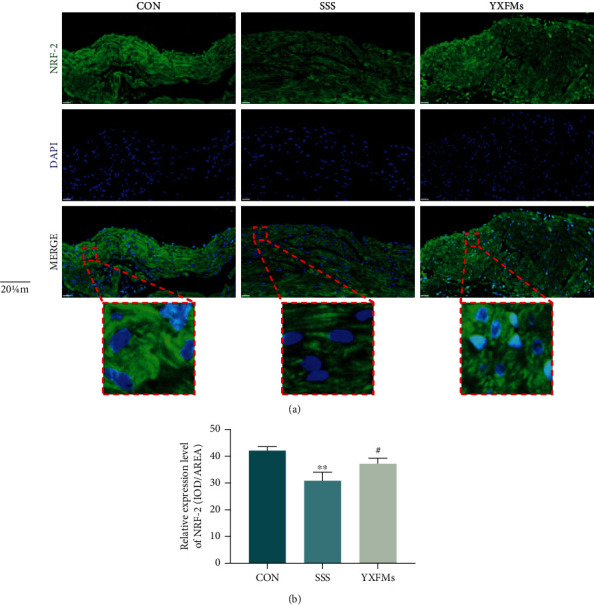
YXFMs alleviate NRF-2 expression in SSS mice. (a) Immunofluorescence staining for NRF-2 expression assay (scale = 20 *μ*m). (b) Comparative analyses for immunofluorescence staining. ^∗∗^*P* < 0.01 in comparison to the CON group, ^#^*P* < 0.05 in comparison to the SSS group. CON: control group; SSS: sick sinus syndrome group; YXFMs: Yixin-Fumai granule-administered group.

NRF-2 is the key protein in the NRF-2/HO-1 pathway. After NRF-2 is activated, it can dissociate from KEAP-1, transfer from the cytoplasm to the nucleus, and bind to AREs to promote HO-1 transcription, resulting in various antioxidant effects, including ROS scavenging. Immunofluorescence staining was used to assay NRF-2 expression in the SAN of our mice; the results showed that green fluorescence in the SAN of the SSS group was significantly lower than that in the SSS of the CON group, suggesting that senescence reduces NRF-2 expression in the SAN (Figures 4(a) and 4(b); ^∗∗^*P* < 0.01). After YXFM administration, green fluorescence in the SAN of the YXFMs group was significantly higher than that in the SAN of the SSS group, and it was transferred from the cytoplasm to the nucleus (Figures 4(a) and 4(b); ^#^*P* < 0.05). This evidence confirmed that YXFMs can effectively promote NRF-2 expression and activation and then inhibit senescence-induced SAN peroxidation and ROS accumulation through the NRF-2/HO-1 pathway.

### 3.4. YXFMs Thwart SHOX2 and CACNA1G Reduction within SSS Mice SAN

**Figure 5:**
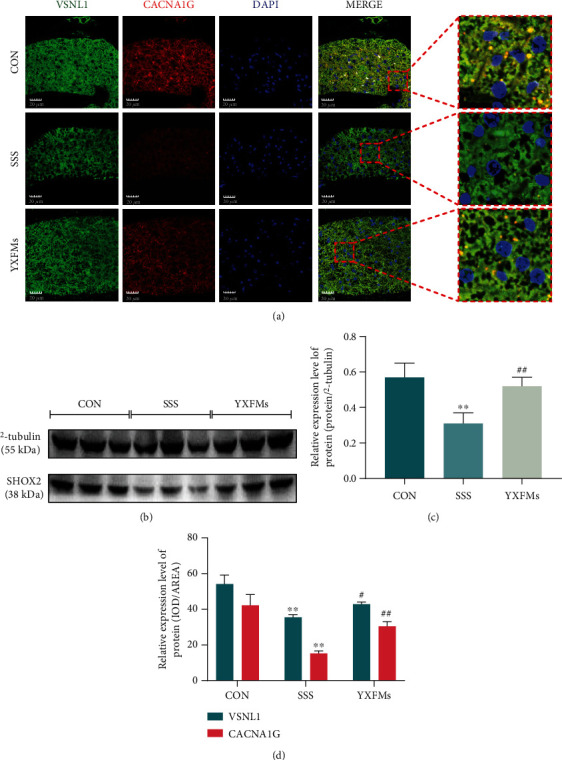
YXFMs thwart CACNA1G reduction within SSS mice SAN. (a) Immunofluorescence staining assay for VSNL1 and CACNA1G (scale = 20 *μ*m). (b) Western blotting assay for SHOX2. (c, d) Comparative analyses for Western blotting results and fluorescence intensities. ^∗∗^*P* < 0.01 in comparison to the CON group, ^##^*P* < 0.01 and ^#^*P* < 0.05 in comparison to the SSS group. CON: control group; SSS: sick sinus syndrome group; YXFMs: Yixin-Fumai granule-administered group.

VSNL1, a protein highly, specifically expressed in the mammalian SAN, is almost not expressed in atrial working cardiomyocytes [34], was used to specifically visualize SAN in immunofluorescence staining experiments, and assess whether P cells in the SAN transformed into working cardiomyocytes; moreover, the normal expression of CACNA1G is the basis of *I*_Ca−T_ produced by P cells in the SAN. We did perform VSNL1/CACNA1G immunofluorescence staining in SAN tissue sections of each mouse group. The results demonstrated that VSNL1 (green fluorescence) and CACNA1G (red fluorescence) in the SAN of the SSS group were decreased extensively (Figures 5(a) and 5(d); ^∗∗^*P* < 0.01), suggesting that the P cells within SSS mice SAN might have been transiting to working cardiomyocytes and decreased pacing function. VSNL1 and CACNA1G expression was considerably higher in YXFMs-treated mice than in the SSS mice (Figures 5(a) and 5(d); ^##^*P* < 0.01, ^#^*P* < 0.05), which may specify that YXFMs inhibit the transition of P cells and improve the pacing function of SAN. In addition, the Western blotting results of the SAN tissues of the mice in each individual group indicated that SHOX2 expression was significantly lower in the SSS group than in the control group, whereas it was significantly higher within the YXFMs group, in comparison to the SSS group (Figures 5(b) and 5(c); ^∗∗^*P* < 0.01, ^##^*P* < 0.01), suggesting that YXFMs promotes SHOX2 expression within SSS mice SAN. This evidence suggests that P cells in the SAN of SSS mice may be suffering from the transformation into working cardiomyocytes, the T-type calcium channel is disappearing, and the pacing function decreases. However, YXFMs treatment can significantly inhibit this process, which may be related to increased SHOX2 and CACNA1G expression.

### 3.5. YXFMs Activate NRF-2 and Improve SHOX2 Deficiency in Senescent HL-1 Cells

**Figure 6:**
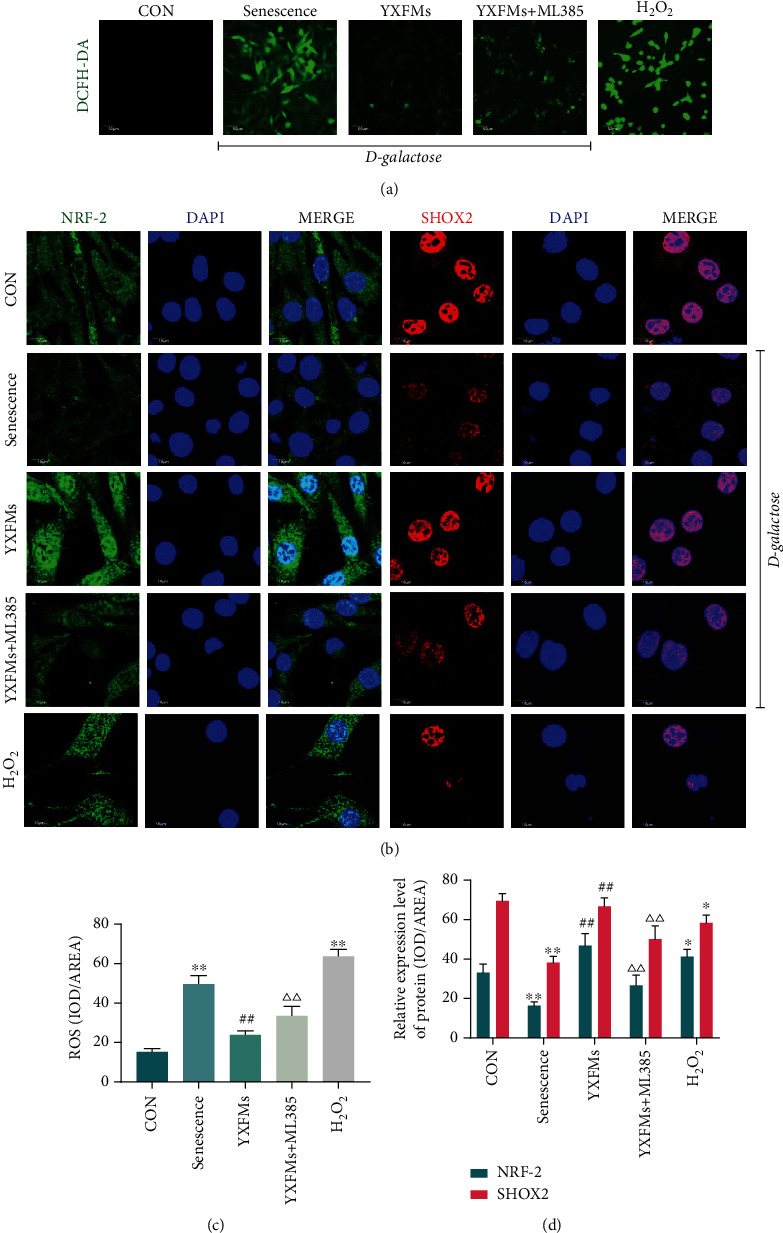
YXFMs activated NRF-2 and improve SHOX2 deficiency in senescent HL-1 cells. (a) DCFH-DA staining for ROS assay (scale = 50 *μ*m). (b) Immunofluorescence staining for NRF-2 and SHOX2 assay (scale = 10 *μ*m). (c) Comparative analyses for ROS content. (d) Comparative analyses for the NRF-2 and SHOX2 expression. ^∗∗^*P* < 0.01 and ^∗^*P* < 0.05 in comparison to the CON group, ^##^*P* < 0.01 in comparison to the senescence group, ^△△^*P* < 0.01 in comparison to the YXFM group. CON: control group; senescence: D-galactose administered group; YXFMs: D-galactose + Yixin-Fumai granule-administered group; YXFMs + ML385: D-galactose + Yixin-Fumai granule + NRF-2 inhibitor ML385-administered group; H_2_O_2_: hydrogen peroxide-administered group.

D-Galactose, a monosaccharide, can be used to induce senescence in animals and cells [35, 36]. Here, we treated hiPSC-AMs with D-galactose to stimulate senescence in P cells. ROS accumulation is an important manifestation of cell senescence, and DCFH-DA is a fluorescent probe which is commonly used for assaying ROS. The results of DCFH-DA assay indicated that the ROS content was elevated within senescent HL-1 cells compared with that within the control group; YXFMs significantly reduced ROS levels in the senescent HL-1 cells (Figures 6(a) and 6(c); ^∗∗^*P* < 0.01, ^##^*P* < 0.01). However, 5 *μ*M ML385 weakened the effect of YXFMs because the ROS content in the HL-1 cells was significantly higher in the YXFMs + ML385 group than that in the YXFMs group (Figures 6(a) and 6(c); ^△△^*P* < 0.01). In other words, YXFMs could reduce ROS accumulation induced by D-galactose through the NRF-2/HO-1 pathway. Furthermore, immunofluorescence staining indicated that senescent HL-1 cells demonstrated the characteristics of NRF-2 and SHOX2 deficiency; however, after YXFMs treatment, NRF-2 and SHOX2 expression recovered (Figures 6(b) and 6(d); ^∗∗^*P* < 0.01, ^##^*P* < 0.01). In other words, senescence can induce the loss of NRF-2 and SHOX2 expression, and YXFMs can inhibit this process. However, ML385 reversed the effect of YXFMs because the expression of NRF-2 and SHOX2 in the HL-1 cells was considerably lower than that in the YXFMs + ML385 group and that in the YXFMs group (Figures 6(b) and 6(d); ^△△^*P* < 0.01). In other words, ROS scavenging by YXFMs may depend on NRF-2 regulation and SHOX2 expression may be regulated by NRF-2 and ROS.

To further investigate the relationship between NRF-2, ROS, and SHOX2, we treat HL-1 cells with 200 *μ*M H_2_O_2_ and found that compared with the CON group, exogenous oxidative stress induced by H_2_O_2_ activated endogenous antioxidant mechanisms: NRF-2 as well as oxidative stress increased the ROS content significantly (Figures 6(a)–6(d); ^∗^*P* < 0.05, ^∗∗^*P* < 0.01) but reduced SHOX2 expression considerably (Figures 6(a)–6(d); ^∗^*P* < 0.05); this result indicated that ROS may hinder SHOX2 expression, that ROS scavenging may contribute to the normal SHOX2 expression, and that NRF-2 possibly regulates SHOX2 via ROS scavenging.

This evidence suggests that D-galactose can effectively led to cell senescence, reduce NRF-2 expression, and cause ROS accumulation, in turn resulting in decreased SHOX2 expression. This mechanism has not been reported thus far. YXFMs may inhibit ROS accumulation by promoting NRF-2 expression and thereby increase SHOX2 expression to improve SAN function.

### 3.6. YXFMs Improve Pulsatile Dysfunction in Senescent hiPSC-AMs

**Figure 7:**
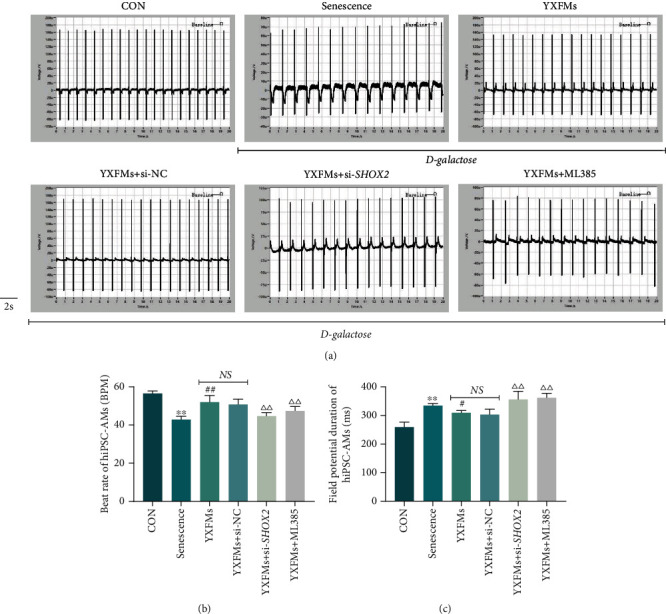
YXFMs improve pulsatile dysfunction in senescent hiPSC-AMs. (a) Field potential traces of hiPSC-AMs. (b, c) Comparative analyses for the beat rate and field potential duration of hiPSC-AMs. ^∗∗^*P* < 0.01 in comparison to the CON group, ^##^*P* < 0.01 and ^#^*P* < 0.05 in comparison to the senescence group, ^△△^*P* < 0.01 in comparison to the YXFM group; NS: no statistical significance; CON: control group; senescence: D-galactose administered group; YXFMs: D-galactose + Yixin-Fumai granule-administered group; YXFMs + si-NC: D-galactose + Yixin-Fumai granule-administered + si-*NC* transfection group; YXFMs + si-*SHOX2*: D-galactose + Yixin-Fumai granule-administered + si-*SHOX2* transfection group; YXFMs + ML385: D-galactose + Yixin-Fumai granules + NRF2 inhibitor ML385-administered group.

To verify the influence of senescence on the SAN function and the role of YXFMs *in vitro*, hiPSC-AMs were treated with D-galactose so as to simulate senescent SAN P cells, and electrophysiological assays were performed on them. Results of CardioExcyte 96 have established that in comparison with that of the CON group, the deteriorated beat rate and field potential duration were seen in the senescence group (Figures 7(a)–7(c); ^∗∗^*P* < 0.01); in other words, senescent hiPSC-AMs demonstrated pulsatile dysfunction. Moreover, in the YXFMs group, the beat rate was relatively fast and the field potential duration was relatively short (Figures 7(a)–7(c); ^##^*P* < 0.01, ^#^*P* < 0.05). However, after incubation with the NRF-2 inhibitor ML385, the effects of YXFMs were cancelled; the hiPSC-AMs in the YXFMs + ML385 group demonstrated a relatively slow beat rate and relatively long field potential duration (Figures 7(a)–7(c); ^△△^*P* < 0.01). This result suggested that YXFMs improved pulsatile dysfunction in senescent hiPSC-AMs and that this effect is related to the NRF-2/HO-1 pathway; combined with the results of DCFH-DA assay and immunofluorescence staining in HL-1 cells, this effect may originate from NRF-2/HO-1 pathway-mediated SHOX2 activation. To verify this, we transfect si-*SHOX2* into senescent hiPSC-AMs treated with YXFMs and found that *SHOX2* silencing abrogated the protective effects of YXFMs; hiPSC-AMs in the YXFMs + si-*SHOX2* group showed pulsatile dysfunction again (Figures 7(a)–7(c); ^△△^*P* < 0.01); in other words, NRF-2/HO-1 pathway-mediated SHOX2 activation is key in the YXFMs-induced improvement in the pulsatile function of senescent hiPSC-AMs. However, how SHOX2 affects pulsatile function remains unknown; to address this question, the BMP4 activator SB4, the BMP4 inhibitor LDN193189, and the T-type calcium channel inhibitor Mibefradil were applied in the subsequent experiments.

### 3.7. YXFMs Improve Pulsatile Function in SHOX2-Silenced hiPSC-AMs

**Figure 8:**
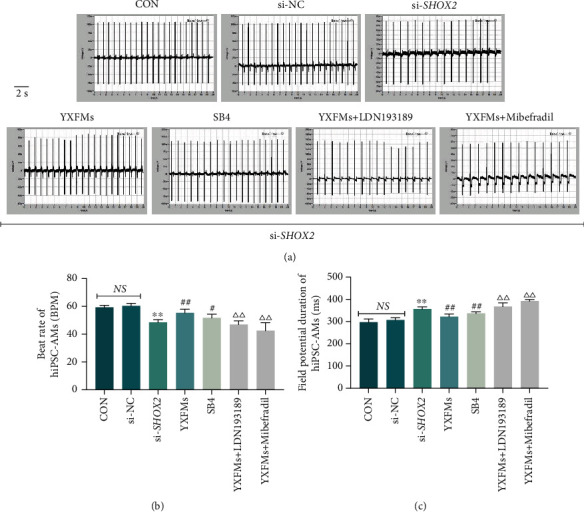
YXFMs improve pulsatile function in SHOX2-silenced hiPSC-AMs. (a) Field potential traces of hiPSC-AMs. (b, c) Statistical results for the beat rate and field potential duration of hiPSC-AMs. ^∗∗^*P* < 0.01 in comparison to the si-NC group, ^##^*P* < 0.01 and ^#^*P* < 0.05 in comparison to the si-*SHOX2* group, ^△△^*P* < 0.01 in comparison to the YXFM group; NS: no statistical significance; CON: control group; si-NC: si-NC transfection group; si-*SHOX2*: si-*SHOX2* transfection group; YXFMs: si-*SHOX2* transfection + Yixin-Fumai granule-administered group; SB4: si-*SHOX2* transfection + BMP4 activator SB4-administered group; YXFMs + LDN193189: si-*SHOX2* transfection + Yixin-Fumai granule + BMP4 inhibitor LDN193189-administered group. YXFMs + mibefradil: si-*SHOX2* transfection + Yixin-Fumai granule + T-type calcium channel inhibitor mibefradil-administered group.

hiPSC-AMs were divided into CON, si-NC, si-*SHOX2*, YXFM, SB4, YXFMs + LDN193189, and YXFMs + mibefradil groups, and the corresponding administrations were performed. The CardioExcyte 96 results showed no significant differences in the beat rate and field potential duration between the si-NC and CON groups (Figures 8(a)–8(c); ^NS^*P* > 0.05). However, compared with the si-NC group, the beat rate of cells in the si-*SHOX2* group decreased significantly and the field potential duration was extended (Figures 8(a)–8(c); ^∗∗^*P* < 0.01). After YXFMs or BMP4 activator SB4 treatment, the beat rate of hiPSC-AMs increased significantly and the field potential duration shortened (Figures 8(a)–8(c); ^##^*P* < 0.01, ^#^*P* < 0.05); however, the BMP4 inhibitor LDN193189 cancelled the protective effect of YXFMs, and compared with the YXFMs group, hiPSC-AMs in the YXFM + LDN193189 group showed pulsatile dysfunction, with a lower beat rate and longer field potential duration (Figures 8(a)–8(c); ^△△^*P* < 0.01). BMP4 is a downstream target of SHOX2. The BMP4 inhibitor LDN193189 cancelled the effect of YXFMs, and as a contrast, BMP4 activator SB4 activated BMP4 and produced pulsatile function protective effect for hiPSC-AMs in the presence of *SHOX2* gene silencing. In other words, the SHOX2/BMP4 axis may be involved in the regulation of hiPSC-AM pulsatile function by YXFMs. In addition, the application of the T-type calcium channel inhibitor mibefradil also abolished the protective effect of YXFMs in hiPSC-AMs (Figures 8(a)–8(c); ^△△^*P* < 0.01), suggesting that T-type calcium channels may be the target ion channels of YXFMs; however, further verification is warranted.

### 3.8. YXFMs Increase CACNA1G Expression through SHOX2 Upregulation

**Figure 9:**
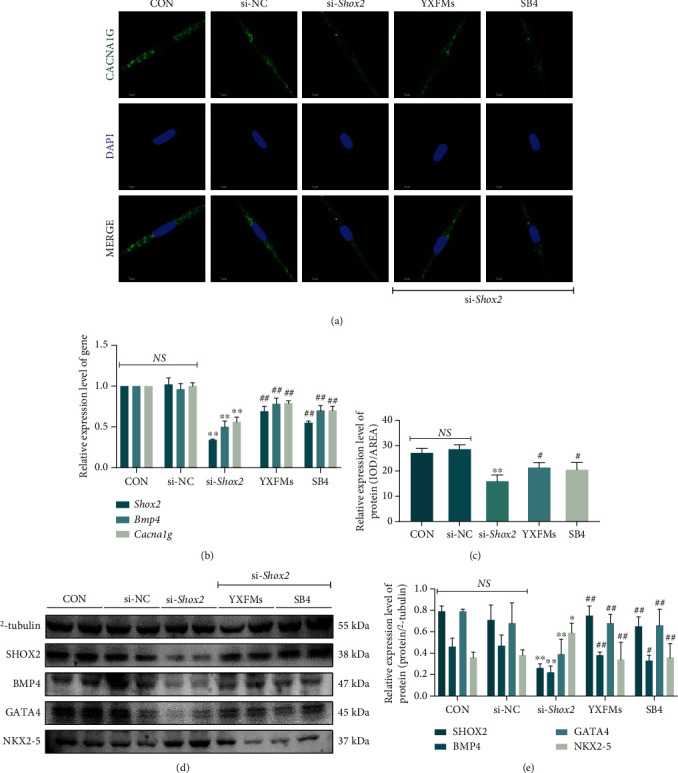
YXFMs increase CACNA1G expression through SHOX2 upregulation. (a) CACNA1G expression in HL-1 cells (scale = 10 *μ*m). (b) Comparative analyses for RT-qPCR. (c) Comparative analyses for immunofluorescence staining. (d) Western blotting assay for SHOX2, BMP4, GATA4, and NKX2-5 in HL-1 cells. (e) Comparative analyses for Western blotting. ^∗∗^*P* < 0.01 and ^∗^*P* < 0.05 in comparison to the si-NC group, ^##^*P* < 0.01 and ^#^*P* < 0.05 in comparison to the si-*Shox2* group. NS: no statistical significance; CON: control group; si-NC: si-NC transfection group; si-*Shox2*: si-*Shox2* transfection group; YXFMs: si-*Shox2* transfection + Yixin-Fumai granule-administered group; SB4: si-*Shox2* transfection + BMP4 activator SB4-administered group.

HL-1 cells were divided into CON, si-NC, si-*Shox2*, YXFMs, and SB4 groups, and the corresponding administrations were performed. The RT-qPCR results demonstrated no significant difference in *Shox2*, *Bmp4*, and *Cacna1g* expression between the si-NC and CON groups (Figure 9(b); ^NS^*P* > 0.05). However, *Shox2*, *Bmp4*, and *Cacna1g* expression was markedly reduced within the si-*Shox2* group in comparison to the si-NC group (Figure 9(b); ^∗∗^*P* < 0.01). *Shox2*, *Bmp4*, and *Cacna1g* expression in cells treated with YXFMs or the BMP4 agonist SB4 increased significantly (Figure 9(b); ^##^*P* < 0.01). Consequently, YXFMs may promote *Cacna1g* expression by regulating *Shox2* and *Bmp4* expression. Next, the immunofluorescence staining was used as a way to assay CACNA1G expression in the membrane of HL-1 cells. The results showed that compared with the si-NC group, the fluorescence intensity within the si-*Shox2* group was markedly reduced but though was promoted within YXFMs/SB4 groups (Figures 9(a) and 9(c); ^#^*P* < 0.05). Western blotting is a process that is used next to assay the expression of proteins relating to *Shox2*, and the results showed no difference between the CON and si-NC groups. However, SHOX2, BMP4, and GATA4 expression in the si-*Shox2* group cells was decreased accompanied by increased NKX2-5 expression (Figures 9(d) and 9(e); ^∗∗^*P* < 0.01, ^∗^*P* < 0.05); moreover, SHOX2, BMP4, and GATA4 expression recovered significantly after YXFMs or SB4 administration and the expression of NKX2-5 also decreased significantly (Figures 9(d) and 9(e); ^##^*P* < 0.01, ^#^*P* < 0.05). These results suggested that YXFMs promote CACNA1G expression and improve the T-type calcium channel through the SHOX2/BMP4/GATA4/NKX2-5 axis.

### 3.9. Network Pharmacology and Molecular Docking

**Figure 10:**
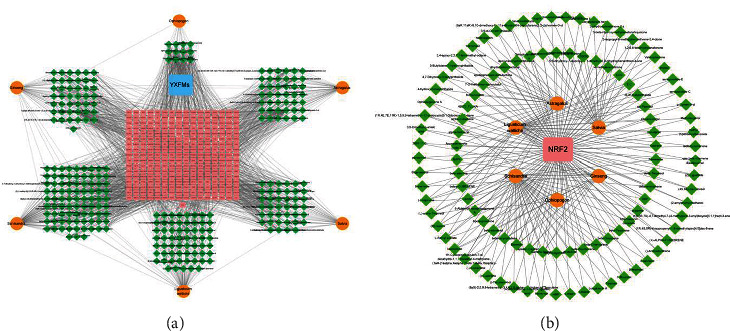
Network pharmacological result. (a) YXFM–herb–compound–target network. (b) Herb–compound–NRF-2 network. Orange, green, and pink represent herbs, compounds, and targets, respectively.

**Figure 11:**
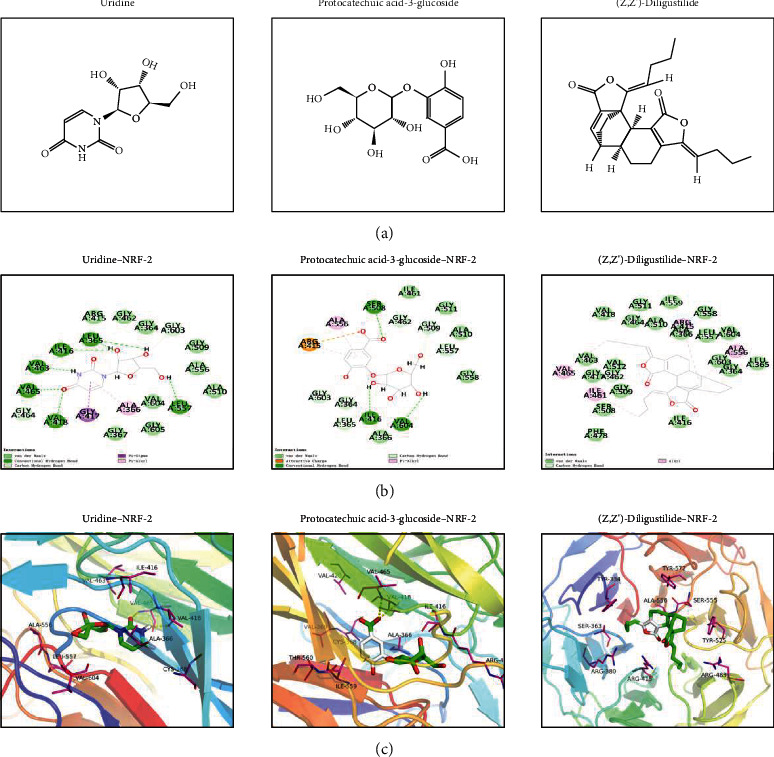
Molecular docking result. (a) Structural formula of compounds. (b, c) 2D and 3D display of compound–NRF-2 molecular docking.

After searching and screening the BATMAM-TCM database, we obtained 282 compounds and 273 potential drug targets of YXFMs. Cytoscape 3.7.2 was used to create YXFMs–herb–compound–target interaction networks, with 562 nodes and 2116 edges (Figure 10(a)). Then, NRF-2 was selected to create the herb–compound–NRF-2 interaction network, with 114 nodes and 234 edges (Figure 10(b)). Next, cytoHubba was used to calculate the degree value of compounds interacting with NRF-2 and the top 3 compounds were uridine, protocatechuic acid-3-glucoside, and (Z,Z′)-diligustilide. These components were selected for molecular docking with NRF-2, and the results showed that all of these compounds could form stable docking with NRF-2 (bonding energy < 5.0 kcal · mol^−1^). In addition, Discovery Studio 2019 was used to dock selected compounds with NRF-2 and calculate the LibDockScore; the results indicated that all the selected compounds could dock with NRF-2 semiflexibly and docking sites were discovered as well. However, the domain 6TYP of NRF-2 could only dock with protocatechuic acid-3-glucoside and (Z,Z′)-diligustilide with LibDockScore > 100 (Table 3). Considering the RMSD, chemical energy, and docking score, the (Z,Z′)-diligustilide–NRF-2 docking body was the most stable. Finally, PyMOL and Discovery Studio 2019 were used to output the molecular docking results in 2D and 3D (Figures 11(b) and 11(c)).

## 4. Discussion

SSS incidence is related to age: older population is at a higher risk. In SSS pathogenesis, SAN dysfunction due to degenerative structural changes play an important role [37, 38]. SAN dysfunction is often characterized by the structural remodel: SAN fibrosis, ROS accumulation, and the transformation of P cells into working cardiomyocytes; it causes ion channel remodeling and electrical conduction delay, which leads to decreased *Hcn4* and *Cacna1g* expression, followed by a decline in the pacing function of SAN [39, 40]. HCN4 channel-mediated current *I*_f_ and T-type calcium channel-mediated current *I*_Ca−T_ are key for maintaining the phase-4 automated depolarization of P cells within SAN. Under physiological conditions, the HCN4 channel and T-type calcium channel are distributed upon P cell-membrane transport extracellular cations into cells when activated at approximately −70 mV and raise the membrane potential to approximately −40 mV. Next, the L-type calcium channel-mediated current *I*_Ca−L_ initiates the phase-0 depolarization of P cells, whereas in the senescent SAN, *I*_f_, *I*_Ca−T_, and *I*_Ca−L_ are obviously attenuated [41].

Many studies have shown that senescence can lead to SAN dysfunction and decreased SAN function in animals, such as zebrafish and guinea pigs [42–44]. In the current study, SSS mice had a worsened heart rate and R-R intervals, along with the presence of a sinoatrial block, severe fibrosis, ROS accumulation, and NRF-2 expression deficiency in the SAN. In addition, the expression of CACNA1G, a key component of T-type calcium channels, was significantly decreased in the SAN of SSS mice and the expression of VSNL1, used to assess whether P cells in the SAN transform into working cardiomyocytes, was also decreased. VSNL1 specifically was expressed in the SAN with large quantities, and it was almost not expressed in atrial working cardiomyocytes [34]. Decreased heart rate, increased R-R intervals, severe SAN fibrosis, ROS accumulation, and decreased VSNL1, CACNA1G, and NRF-2 expression indicated that the SAN of the SSS mice may demonstrate degenerative changes, with the P cells in the SAN transforming into working cardiomyocytes; this process may be related to ROS accumulation due to NRF-2 inactivation. The Western blotting results also demonstrated low SHOX2 expression within SSS mice SAN; SHOX2 is an important transcription factor which regulates SAN differentiation, ensures the normal existence of P cells, and prevents its metaplasia to working cardiomyocytes. This evidence suggests that in the SAN of SSS mice, P cells may transform into working cardiomyocytes due to NRF-2 deficiency-induced ROS accumulation and lack of SHOX2, thus leading to T-type calcium channel dysfunction and the resulting SAN dysfunction.

YXFMs, a marketed traditional Chinese medication used to treat bradyarrhythmia, have a strong antioxidant effect. In a previous study, we found that the effect of YXFMs in SSS depends on NRF-2/HO-1 pathway regulation and HCN4 expression. In the current study, we focused on YXFMs' regulation function in T-type calcium channels and explored its relationship with the NRF-2/HO-1 pathway and SHOX2.

The results of the *in vivo* experiments demonstrated that YXFMs can cause a significant increase in the heart rate of SSS mice, reduce R-R intervals, inhibit SAN fibrosis and ROS accumulation, and promote NRF-2, SHOX2, CACNA1G, and VSNL1 expression in the SAN. This evidence suggested that YXFMs may be able to inhibit the transformation of P cells into working cardiomyocytes by inhibiting ROS accumulation in the SAN through NRF-2 activation, thereby upregulating SHOX2 expression and promoting T-type calcium channel expression and thus improving SAN function. To verify this, we treated HL-1 cells with D-galactose to simulate senescent P cells and found that it increased ROS levels, accompanied by a decrease in NRF-2 and SHOX2 expression; YXFMs intervened in this process, inhibiting the increase in ROS levels and the loss of NRF-2 and SHOX2 expression. However, the NRF-2 inhibitor ML385 cancelled the NRF-2-activation effect of YXFMs and senescent HL-1 cells showed ROS accumulation accompanied by decreased SHOX2 expression. Next, we treated HL-1 cells with H_2_O_2_ and found that exogenous oxidative stress induced by H_2_O_2_ activated NRF-2 and increased ROS but also reduced SHOX2 expression. This evidence, for the first time, demonstrated that ROS accumulation may hinder SHOX2 expression and that ROS scavenging caused by NRF-2/HO-1 pathway activation may promote SHOX2 expression.

To verify this result, we treated hiPSC-AMs with D-galactose to simulate senescent P cells by using CardioExcyte 96 and found that senescent hiPSC-AMs demonstrated pulsatile dysfunction and that YXFMs shortened the field potential duration and increased the beat rate considerably. However, this effect was blocked by the NRF-2 inhibitor ML385 or by the *SHOX2* gene interference sequence, suggesting that senescence induces SHOX2 deficiency and pulsatile dysfunction in cardiomyocytes; therefore, YXFMs may be able to inhibit this by upregulating SHOX2 expression through the NRF-2/HO-1 pathway.

To further investigate how SHOX2 affects the pulsatile function of cardiomyocytes, we next transfected si-*SHOX2* and si-*Shox2* into hiPSC-AMs and HL-1 cells, respectively. The results indicated that *SHOX2*-silenced hiPSC-AMs demonstrated longer filed potential duration and lower beat rate; nevertheless, YXFMs significantly reversed this trend and improved the field potential duration and beat rate of *SHOX2*-silenced hiPSC-AMs. However, the BMP4 inhibitor LDN193189 and the T-type calcium channel inhibitor mibefradil cancelled the effects of YXFMs. In contrast, the BMP4 activator SB4 has an effect similar to that of YXFMs in that it improved the pulsatile function of *SHOX2*-silenced hiPSC-AMs. This evidence suggests that BMP4 and T-type calcium channels may be the more downstream targets of SHOX2 as well as the targets of YXFMs. Moreover, the RT-qPCR, Western blotting, and immunofluorescence staining results of HL-1 cells indicated that YXFMs could considerably promote SHOX2, BMP4, and CACNA1G expression in *Shox2*-silenced HL-1 cells, while increasing GATA4 expression and reducing NKX2-5 expression. The activation of GATA4, the downstream regulatory target of BMP4, inhibits NKX2-5 expression and ultimately promotes the expression of pacing-related ion channels including T-type calcium channel expression and prevents P cell transformation into working cardiomyocytes [14, 20].

**Figure 12:**
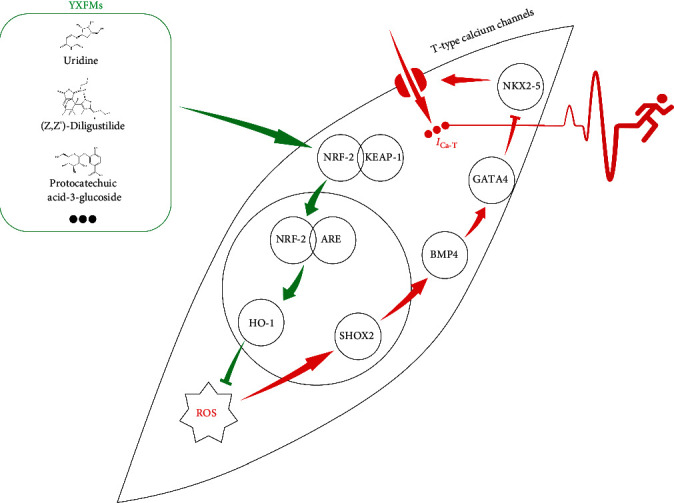
The mechanism of YXFMs improving SAN dysfunction: NRF-2/HO-1 pathway-mediated SHOX2 activation is a key switch.

In conclusion, YXFMs can activate the NRF-2/HO-1 pathway to resist oxidative stress in senescent cells, reduce ROS accumulation, and then promote the expression of SHOX2. SHOX2 in turn regulates CACNA1G expression through downstream BMP/GATA4/NKX2-5 and improves the function of T-type calcium channels, thus preventing the transformation of P cells into working cardiomyocytes and ameliorating diminished SAN function (Figure 12).

Considering SAN improvement due to YXFMs, the effective components that increase the efficacy and reduce the toxicity of YXFMs were analyzed. GO analysis found that YXFMs mainly act on various ion channels in a previous study, but components that can directly interfere with ion channels usually might have an arrhythmia risk. Therefore, we analyzed the compounds that act on NRF-2 to regulate the SAN function from the upstream of ion channels. We screened out compounds that interact with NRF-2 in the YXFMs–herb–compound–target network and analyzed the binding degree of compounds with NRF-2 via molecular docking. The results showed that (Z,Z′)-diligustilide, a phthalides compound from the Chinese herb *L. wallichii*, had the most stable docking with NRF-2. Thus, in the future, we may further verify the (Z,Z′)-diligustilide–NRF-2 interaction to determine a highly effective compound for SSS treatment.

## Figures and Tables

**Table 1:** siRNA target sequences.

si-RNA	Species	Target sequence
si-*Shox2*	Mouse	CACTATCCAGACGCTTTCA
si-*SHOX2*	Human	TCAACTCCATAAAGGTGTT

**Table 2:** RT-qPCR primers.

Gene	Species	Forward primer (5′–3′)	Reverse primer (5′–3′)
*Shox2*	Mouse	CCCTGGAACAACTCAACGA	ATGACTATCCTGCTGAAATGG
*Bmp4*	Mouse	CGTAGTCCCAAGCATCA	ATCAGCCAGTGGAAAGG
*Cacna1g*	Mouse	GCCATTGTCACTGTCTTTCA	CCGTTTGCCGATTTCCT
*β-Actin*	Mouse	CTGTGCCCATCTACGAGGGCTAT	TTTGATGTCACGCACGATTTCC

**Table 3:** Molecular docking results.

Structural domain	Compound	Vina	RMSD	LibDockScore	Hydrogen bond interaction	Hydrophobic interaction
NRF2 (6TYP)	Uridine	−7	1.884	95.0622	LEU:365,ILE:416,VAL:463,VAL:465,GLY:464,VAL:418,LEU:557,GLY:603	GLY:417,ALA:366
NRF2 (6TYP)	Protocatechuic acid-3-glucoside	−7.7	1.392	106.892	SER:508,GLY:603,GLY:364,LEU:365,ILE:416,VAL:604,GLY:509,GLY:462,SER:508	ALA:556,ARG:415
NRF2 (6TYP)	(Z,Z′)-Diligustilide	−8.7	2.038	119.958	GLY:462	VAL:465,ILE:461,ALA:556,ARG:415

## Data Availability

The data used to support the findings of this study are available from the corresponding author upon request.

## Supplementary Materials

Supplementary Materials Supplementary files contains editorial certificate and analysis certificate for references.
