# Supplementary material for: NRF-2/HO-1 Pathway-Mediated SHOX2 Activation Is a Key Switch for Heart Rate Acceleration by Yixin-Fumai Granules
Source: Oxid Med Cell Longev. 2022 Sep 26;2022:8488269. doi: 10.1155/2022/8488269 (PMC9529460; doi:10.1155/2022/8488269)
Supplement: Supplementary Materials — Supplementary files contains editorial certificate and analysis certificate for references. [file 8488269.f1.zip › Analysis certificate (1).pdf]

Cardiomyocytes CERTIFICATE OF ANALYSIS

PRODUCT: Custom Service
NOTE: H-7
DATE OF REPORT: 10. Jan. 2022

PRODUCT DESCRIPTION
Cardiomyocytes are human iPSC-derived cardiomyocytes suitable for drug screening, toxicity evaluation and other *in vitro* applications.

Viability

TEST DESCRIPTION	SPECIFICATION	RESULT
Cell Number	⩾2×106 cells	**2.18×106 cells**
Post-Thaw Recovery	>70% adhere	**Pass**

Sterility

TEST DESCRIPTION	SPECIFICATION	RESULT
Bacteria	Negative	**Pass**
Fungi (Yeast and Mold)	Negative	**Pass**
Mycoplasma	Negative	**Pass**

Characterization

PLURIPOTENCY MARKERS	SPECIFICATION	RESULT
Cardiac Troponin T (cTnT)	>70% cTnT	**97.8%**

Brightfield

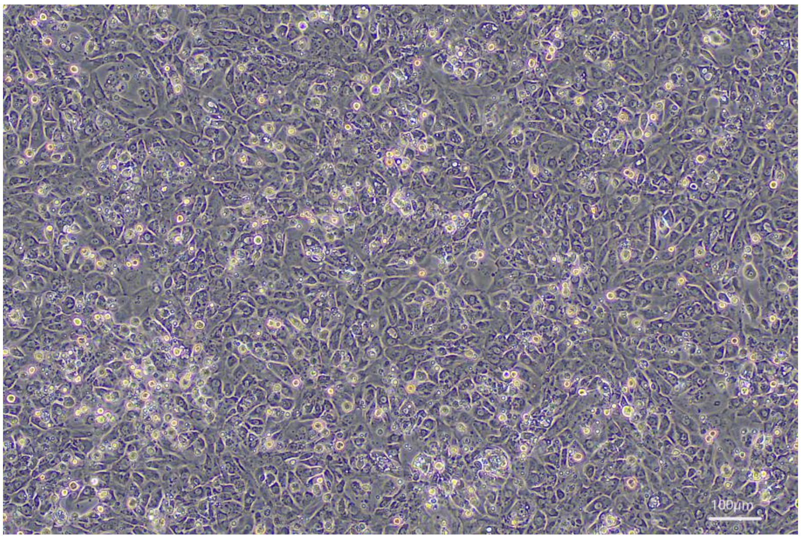

Scale bar 100um

Flow cytometry analysis of cardiomyocyte marker cTnT and MLC-2a expression in Cardiomyocytes.

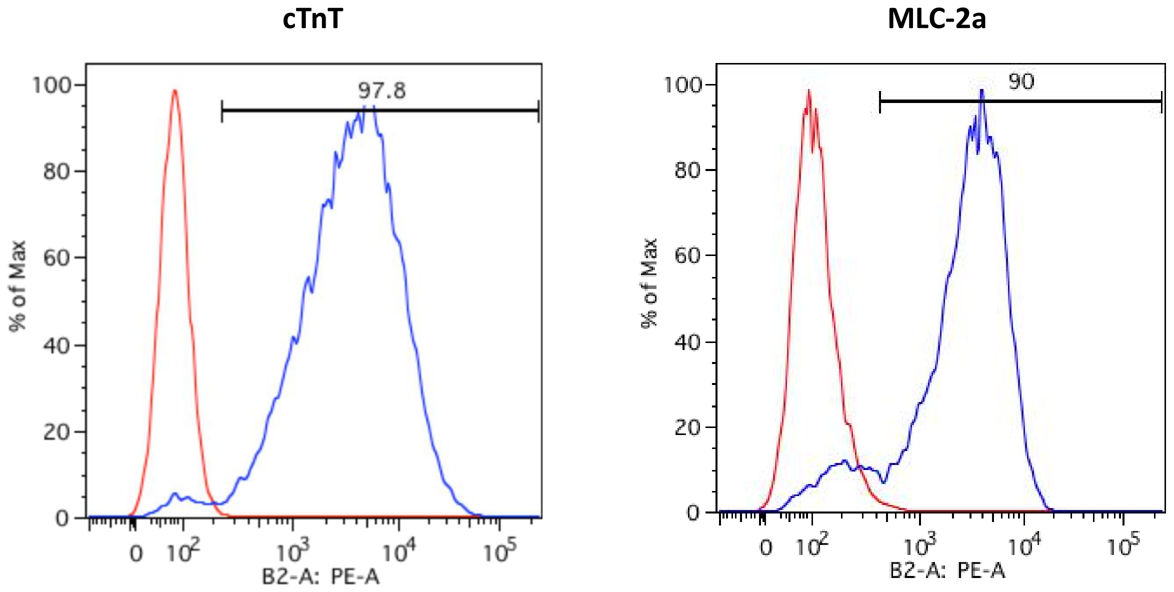

Immunofluorescence analysis of cTnT expression in Cardiomyocytes.

Cardiomyocytes were labeled for cTnT(Red). Nuclei were labeled with DAPI (Blue).

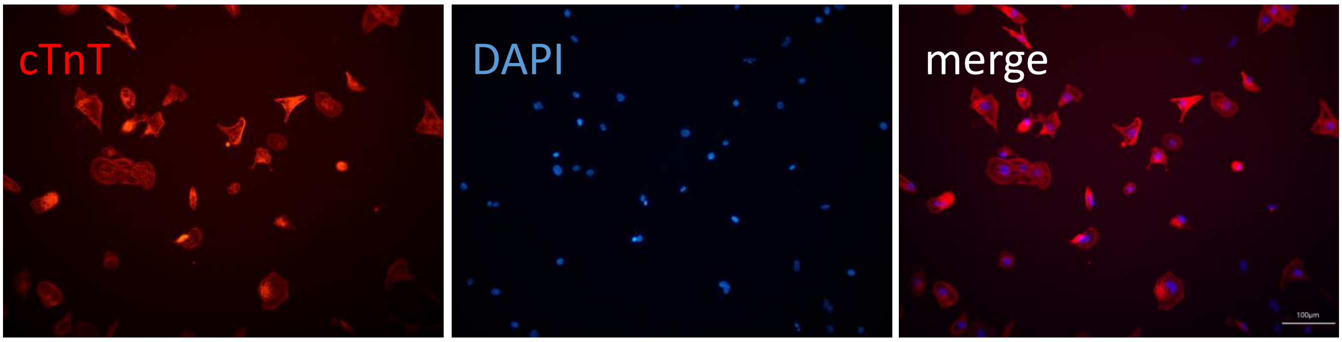

Scale bar 100um
