# Supplementary material for: NRF-2/HO-1 Pathway-Mediated SHOX2 Activation Is a Key Switch for Heart Rate Acceleration by Yixin-Fumai Granules
Source: Oxid Med Cell Longev. 2022 Sep 26;2022:8488269. doi: 10.1155/2022/8488269 (PMC9529460; doi:10.1155/2022/8488269)
Supplement: Supplementary Materials — Supplementary files contains editorial certificate and analysis certificate for references. [file 8488269.f1.zip › Editorial certificate (1).pdf]

https://www.mjeditor.com

EDITORIAL CERTIFICATE

This document certifies that the manuscript listed below was edited for grammar,punctuation,spelling,and overall style by one or more expert native English speaking editors with a PhD degree.

Manuscript information

ID：MJ21504

Editing date：2022.09.04

Title:NRF-2/HO-1 pathway-mediated SHOX2 activation is a key switch for heart rate acceleration by Yixin-Fumai granules

Author(s) : Heng Zhang, Chen Chen, Yue Liu, Lu Ren, Jing Qi, Yang Yang, Wei Chen, Yingjia Yao, Xintong Cai, Zhuang Liu, Miao Hao, Lingkang Li, Zisu Deng, Mingyu Sun, Yongping Lu, Keyan Chen, Ping Hou

Recommendation after language editing

☑Submitting to target journal directly
☐Submitting to target journal after minor revisionz
☐Re-editing required after major revision
☐Not suitable for publication

Certificate by

Sophiya.K

Editor in Chief

MJ Language Editing Services,Shenzhen,China

Disclaimer:Our service does not involve authenticity review or ethical review on the data(including images)presented in the manuscript.Neither the research content nor the author's intentions were altered in any way during the editing process.Documents receiving this certification should be English-ready for publication.The authors have the option to accept or reject our suggestions and changes in the edited document.However,we do not bear responsibility for revisions made to the document after our editing.If the manuscript is suspected of plagiarism,please contact the authors in time.

MJ Language Editing Services
Diwang Building, No. 5002 Shennan Road, Luohu District, Shenzhen, China
Tel:+086 0755 25100506
